# Spatial pattern separation deficits in early Alzheimer’s disease are comparable in humans and animal models

**DOI:** 10.1038/s41598-026-36266-y

**Published:** 2026-01-22

**Authors:** Martina Laczó, Kristyna Maleninska, Natalie Khazaalova, Sarka Borovska, Martin Vyhnalek, Jakub Hort, Ales Stuchlik, Jan Svoboda, Jan Laczó

**Affiliations:** 1 Department of Neurology, Second Faculty of Medicine, Charles University, Motol and Homolka University Hospital, Prague, Czechia; 2 Laboratory of Neurophysiology of Memory, Institute of Physiology of the Czech Academy of Sciences, Prague, Czechia; 3 Center for Advanced Studies of Brain and Consciousness, National Institute of Mental Health, Klecany, Czechia

**Keywords:** Amyloid beta, Biomarkers, Memory, Morris water maze, Spatial learning, Translational test, Neurology, Neuroscience

## Abstract

Spatial pattern separation (SPS) is a memory process that enables the discrimination of similar spatial locations. This process is vulnerable to pathophysiological changes in the early stages of Alzheimer’s disease (AD), but the translational potential of its testing remains unclear. This study aimed to evaluate the potential of SPS testing as a translational cognitive marker for identifying early AD and enabling direct comparisons of cognitive outcomes in animals and humans. We used a validated SPS task to examine biomarker-defined participants with amnestic mild cognitive impairment due to AD (AD aMCI; *n* = 56) and cognitively normal (CN) participants (*n* = 60). An animal version of this task, based on a modified Morris Water Maze task, was used to test six-month-old transgenic TgF344-AD rats (*n* = 38) and wild-type (WT) rats (*n* = 36). AD aMCI participants performed worse than CN participants, with performance declining as distance decreased. These results remained unchanged when adjusted for memory performance. TgF344-AD rats performed worse than WT rats in a probe trial with a 90° SPS design, but not in probe trials with an 180° SPS design or no SPS demands. The discriminatory power of the task was similar in the human and animal experiments. The findings demonstrate comparable SPS deficits in the early stages of AD in both humans and rodent models, which are not attributable to general memory impairment. SPS testing enables direct comparisons to be made between the cognitive performance of rats and humans, making it a promising approach for translational AD research.

**Supplementary Information:**

The online version contains supplementary material available at 10.1038/s41598-026-36266-y.

## Introduction

Alzheimer’s disease (AD) is the most common neurodegenerative disease and the leading cause of cognitive impairment in older adults^1^. With the advent of disease-modifying therapies for AD, which may have a significant impact on disease management^2–4^, it is increasingly important to introduce digital tools that can reliably detect subtle cognitive deficits at a very early stage and capture cognitive changes associated with disease progression and treatment effects. These tools should also allow direct comparison of human and animal cognitive performance using similar test designs^5^, providing a more reliable basis for translating the effects of disease-modifying and symptomatic agents on cognitive function from the preclinical to the clinical phase of drug development. In particular, digital spatial memory tests adapted from rodent tasks have proved effective in measuring the effects of AD therapy^6,7^. Among these tools, those that focus on spatial pattern separation may play an important role because they target the functions of the brain regions most affected early in AD^8,9^.

Pattern separation is a key memory process that separates similar and overlapping representations into discrete, non-overlapping representations^10^. It involves discriminating between similar locations, a process known as spatial pattern separation^11^. This process occurs in the hippocampus and is largely influenced by glutamatergic projections from the entorhinal cortex, cholinergic projections from the basal forebrain, and noradrenergic projections from the locus coeruleus^12–15^. The hippocampus, entorhinal cortex, basal forebrain and locus coeruleus are vulnerable to the pathophysiological changes associated with AD, being among the first sites to accumulate neurofibrillary tau^9^. Tau pathology is first observed in the locus coeruleus (corresponding to Braak stage 0), followed by the basal forebrain (corresponding to Braak stages I-II^16^ or potentially stage 0^17,18^, then the entorhinal cortex (Braak stages I-II), and finally the hippocampus (Braak stages II-III), before spreading to other medial temporal lobe and cortical regions (Braak stages IV-VI)^9^. Studies in rodents and humans have demonstrated that lesions or neurodegeneration of these brain regions and their connections lead to impaired spatial pattern separation^19–22^. Indeed, previous clinical research has shown that impaired spatial pattern separation may reflect AD-related atrophy of the posterior hippocampus and posteromedial entorhinal cortex, as well as specific basal forebrain nuclei (e.g., Ch1-2 nuclei), which project to these regions^23^. This research has also suggested that spatial pattern separation testing could serve as a sensitive marker of cognitive changes associated with early AD^23^. Although these findings are promising, there is a lack of translational studies directly comparing the utility of spatial pattern separation tests in early AD and its rodent models.

To address this knowledge gap, we developed an animal version of the Spatial Pattern Separation Task^23^. The aim was to assess the potential of spatial pattern separation testing as a translational cognitive marker for identifying early AD pathophysiology and to allow direct comparison of animal and human cognitive outcomes. Specifically, we assessed the differences in spatial pattern separation performance between biomarker-defined participants with early clinical AD and cognitively normal (CN) older adults, and between transgenic TgF344-AD^24^ and wild-type rats at 6 months of age, as well as the influence of separation distance on spatial pattern separation performance. We hypothesised that AD-related differences in spatial pattern separation would be similar in humans and the animal model. Specifically, the participants with early AD and TgF344-AD rats will perform worse than CN older adults and wild-type rats, respectively, and in both spatial pattern separation performance will vary with separation distance.

## Methods

### Human study

#### Recruitment and inclusion criteria

This study included 116 participants from the Czech Brain Aging Study (CBAS) cohort^25^, comprising 37 men and 79 postmenopausal women. Specifically, participants with amnestic mild cognitive impairment (aMCI) due to AD (AD aMCI; *n* = 56) were recruited at the Memory Clinic of the Charles University, Second Faculty of Medicine, and Motol University Hospital, Prague, Czech Republic. They were referred to the Memory Clinic by general practitioners and neurologists for memory complaints reported by the participants themselves, their informants, or health professionals. CN older adults (*n* = 60) were recruited from the University of the Third Age, senior centres, or were relatives of memory clinic participants and hospital staff. All participants underwent a clinical assessment, routine blood tests, cognitive and spatial pattern separation assessments, and a magnetic resonance imaging (MRI) brain scan. In addition, all participants with aMCI underwent biomarker assessment, including measurement of cerebrospinal fluid (CSF) amyloid-β_1–42_ (Aβ_1–42_), phosphorylated tau_181_ (p-tau_181_), and total tau (t-tau), or amyloid positron emission tomography (PET) imaging, or both^26^. All participants provided written informed consent, and the study, as well as the experimental protocol, were approved by the Ethics Committee of Motol University Hospital (consent number EK701/16). The study and all methods were performed in accordance with the guidelines of the Alzheimer’s Association, regulations of the Czech Medical Association of J. E. Purkyně and Motol University Hospital, and the Declaration of Helsinki.

(i) Participants with AD aMCI (*n* = 56) met the diagnostic criteria for aMCI^27^, which included subjectively perceived memory decline from a previously normal state, objective evidence of memory impairment (i.e., performance > 1.5 standard deviations [SDs] below the mean of the age-, gender- and education-adjusted norms on any memory test), preserved independence in functional abilities (as confirmed by clinical interviews), and the absence of dementia. Participants had a positive AD biomarker profile. Specifically, 39 participants had low levels of CSF Aβ_1–42_, and 32 participants had a positive visual reading of the flutemetamol (18 F) PET scan. Of these, 15 participants had both low CSF Aβ_1–42_ levels and a positive flutemetamol (18 F) PET scan. Participants without confirmed AD biomarker positivity were not included in the study^28^.
(ii) CN participants (*n* = 60) reported no cognitive complaints and had normal performance on standardised cognitive tests, adjusted for age, gender, and education. These participants had no family history of AD or other types of dementia in first-degree relatives. In addition, visual assessment of the MRI scans by a trained cognitive neurologist confirmed the absence of medial temporal lobe atrophy. We introduced these criteria to minimise the risk of including participants who may be at increased risk of AD, such as those with subjective cognitive decline, hippocampal atrophy, or a positive family history of AD^28^.

#### Exclusion criteria

Participants with low visual acuity, severe white matter lesions on MRI (Fazekas score > 2 points), primary brain disorders that may affect cognitive function, including neurological and psychiatric disorders (e.g., Parkinson’s disease, epilepsy, multiple sclerosis, a history of traumatic brain injury or stroke, and a history or current major psychiatric disorder), and a history of alcohol or drug abuse were not included in the study^28^.

#### Spatial pattern separation task

**Fig. 1:**
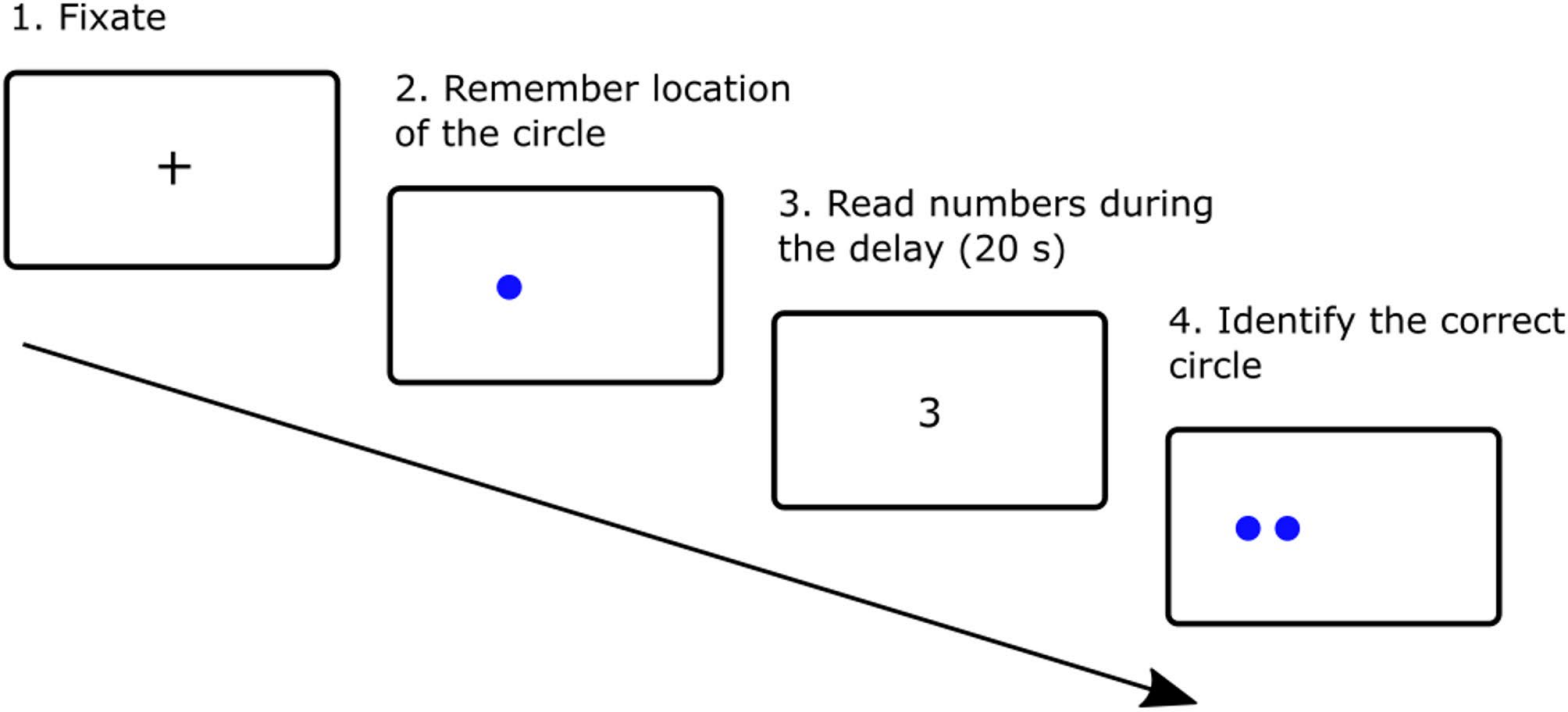
Spatial pattern separation task in humans. An example of one of the 32 trials that participants viewed on a computer screen.

Spatial pattern separation was assessed using a validated Spatial Pattern Separation Task^21,23,29^ (Fig. 1) administered on a 24-inch computer screen. The task consisted of 32 trials, comprising a learning phase followed by a test phase. In the learning phase, participants were instructed to remember the exact location of a blue circle displayed on the screen. This circle, measuring 2 cm in diameter, was presented for 5 seconds at one of 18 possible locations along an invisible horizontal line across the centre of the screen. In the subsequent test phase, two identical blue circles were presented. One of these circles (the target circle) was located in the same position as the original circle in the learning phase (correct choice). The foil circle was situated to the left or to the right of the original position (incorrect choice). The spatial separation distances between the target and foil circles varied across the four conditions: 0 (circle edges touching), 0.5 cm, 1.0 cm, and 1.5 cm. During the test phase, participants were asked to identify the target circle by pressing the green button in their right hand if they thought the target circle was the right one, or by pressing the red button in their left hand if they thought the circle on the left was the target. A 20-second delay separated the learning and test phases. During the delay, participants were instructed to look at the centre of the screen where randomly generated numbers appeared. They were asked to read the numbers aloud to avoid fixating on the location of the target circle. Following the test phase, a small cross appeared in the centre of the screen for 3 seconds to separate the trials. The task consisted of eight trials for each separation distance (0, 0.5, 1.0, and 1.5 cm), totalling 32 trials and lasting 17 minutes. The task was divided into two blocks of 16 trials, with a five-minute break between blocks to minimise participant fatigue. The spatial separation distance and the location of the target circle on a given side (left or right) were pseudo-randomised across the trials^23^. Prior to the task, participants underwent a familiarisation training consisting of six trials. If an error occurred during the training, the experimenter repeated the instructions and training to ensure comprehension of the task.

#### Cognitive assessment

The following tests were used to assess cognitive function: (1) the Rey Auditory Verbal Learning Test (RAVLT) – Trials 1–5 (RAVLT 1–5) and 30-minute Delayed Recall trial (RAVLT-30)^30^ and the Logical Memory – Immediate Recall (LM-IR) and 20-min Delayed Recall (LM-DR) conditions^31^ for verbal memory; (2) the Rey-Osterrieth Complex Figure Test (ROCFT) – the Recall condition after 3 minutes (ROCFT-R) for non-verbal memory^32^; (3) the ROCFT – the Copy condition^32^ and the Clock Drawing Test (CDT)^33^ for visuospatial function; (4) the Trail Making Test (TMT) B^34^ and the Phonemic Verbal Fluency – letters N, K, P for executive function^35^; (5) the Forward and Backward Digit Spans and the TMT A for attention and working memory^31,34^; and (6) the Boston Naming Test – a 30 odd-items version (BNT-30)^36^, and the Categorical Verbal Fluency – Animals for language^35^. The maximum time to complete the TMT A and B was 180 seconds and 300 seconds, respectively, and those who were unable to complete the TMTs in a given time were scored as 181 seconds and 301 seconds, respectively^26^. The self-report Geriatric Depression Scale, a 15-item version^37^, and the Beck Anxiety Inventory^38^ were administered to assess depressive and anxiety symptoms.

#### CSF biomarker analysis

CSF samples were obtained by lumbar puncture in the supine position, collected in 8 mL polypropylene tubes, gently mixed, centrifuged, aliquoted, and stored at −80 °C until analysis. Stored CSF samples were thawed and vortexed before biomarker analysis^26^. CSF collection, processing, and storage procedures followed European guidelines^39^. CSF Aβ_1–42_, p-tau_181_, and t-tau levels were analysed using commercially available enzyme-linked immunosorbent assays (ELISA) (Euroimmun). The cut-off values were set at less than 665 pg/mL for Aβ_1–42_, greater than 48 pg/mL for p-tau_181_, and greater than 358 pg/mL for t-tau^26,40^.

#### Amyloid PET imaging

Dual-phase amyloid PET was used to assess Aβ positivity. PET images were acquired using a Biograph 40 TrueV HD PET/CT scanner (Siemens Healthineers AG). Participants received a single intravenous dose of flutemetamol (18 F; Vizamyl, GE Healthcare). Non-contrast, low-dose computed tomography (CT) brain images were obtained for attenuation correction before the PET scans. A PET list-mode acquisition was performed in two phases: an early (perfusion) and a late (Aβ). The early-phase images were acquired at the time of flutemetamol (18 F) administration for 8 minutes (2 × 4 minutes), and the late-phase images were acquired 90 minutes after flutemetamol (18 F) administration for 10 minutes (2 × 5 minutes)^41^. The flutemetamol (18 F) PET images were visually read as Aβ-positive or Aβ-negative by a board-certified nuclear medicine physician using the GM-EDGE method. Aβ-specific uptake in the grey matter was evaluated in the frontal and lateral temporal lobes, anterior and posterior cingulate cortices, precuneus, striatum, and temporoparietal areas, including the insula^42^.

#### Magnetic resonance imaging

MRI images were acquired on a Siemens Avanto 1.5T scanner (Siemens AG) with a 12-channel phased-array head coil. A high-resolution three-dimensional T1-weighted (3D T1w) Magnetisation-Prepared Rapid Gradient Echo (MPRAGE) sequence was used with the following parameters: TR/TE/TI = 2,000/3.08/1,100 ms, flip angle = 15°, 192 continuous partitions, slice thickness = 1.0 mm, and in-plane resolution = 1 mm^43^. All images were visually inspected by a radiologist to exclude participants with tumours, cortical infarcts, hydrocephalus, or other main brain pathologies. A trained data analyst performed quality control assessments to identify excessive motion artefacts. The 3D T1w images of sufficient quality were available for 96 participants, including CN (*n* = 52) and AD aMCI (*n* = 44) participants^26^.

We utilised a previously published processing pipeline based on a CBAS template to measure volumes of the hippocampus, including the hippocampal head, body, and tail, as well as volumes of the entorhinal cortex, comprising anterolateral and posteromedial subregions, and estimated total intracranial volume (eTIV)^21,23,26^. The skull-stripped 3D T1w images were processed using statistical parametric mapping (SPM8, Wellcome Trust Center for Neuroimaging^44^ and the VBM8-toolbox (http://dbm.neuro.uni-jena.de/vbm/) implemented in MATLAB R202b (MathWorks, Natick, MA). We used a CBAS template based on the manual segmentation of the subregions of the hippocampus and entorhinal cortex, which were aligned in MNI space and derived from 26 cognitively normal older adults recruited from the CBAS^25^. The CBAS template was registered and diffeomorphically warped into each participant’s space using the Advanced Normalization Tools package (http://stnava.github.io/ANTs/). The resulting warp field was used to transform ROI masks of individual hippocampal and entorhinal cortex subregions into the participants’ space. The ROI masks were then masked with a grey matter ROI, and their volumes were extracted. The volumes of the hippocampal body and tail were summed to form the volume of the posterior hippocampus^28^. The volumes from the right and left hemispheres were combined to create a single measure.

The volume of the basal forebrain was measured in accordance with the published protocol^45–47^. MRI data were processed using SPM8 and the VBM8 toolbox, which was implemented in MATLAB R2023b. As in previous studies^21,23,48^, we used a mask of the basal forebrain derived from a cytoarchitectonic map of the basal forebrain cholinergic nuclei, aligned in MNI space, based on histological sections and the MRI scan of a post-mortem brain^44,46^. The mask included basal forebrain subregions corresponding to the Ch1-2, Ch3, Ch4p (posterior), Ch4ai (anterior and intermediate) nuclei and the nucleus subputaminalis. We non-linearly registered images into the MNI152 template and used the resulting DARTEL parameters^49^ to warp the cytoarchitectonic map into individual brain scans. Volumes of the right and left basal forebrain and their subregions were extracted and averaged across both hemispheres. For the statistical analyses, only volumes of the basal forebrain and its Ch1-2 nuclei were utilised, as the cholinergic projections of these nuclei directly target the hippocampus and entorhinal cortex^50^. The volumes were normalised to eTIV using the previously published regression formula^43,51^. The outputs were visually inspected for image and segmentation quality by an experienced reader blinded to clinical and biomarker data^28^.

#### Statistical analysis

Analyses were performed in SPSS (version 28.0, IBM). Statistical significance was set at two-tailed *p* < 0.05. Descriptive characteristics are presented as means and SDs for continuous variables and proportions for categorical variables. *T*-tests and chi-square tests were used to analyse group differences in age, years of education, and gender proportions, respectively. Group differences in cognitive performance were analysed using general linear models (GLM), controlling for age, gender, and years of education. Group differences in volumes of selected brain regions were analysed using GLM, controlling for age and gender. Group differences in spatial pattern separation performance were analysed using linear mixed models (LMM) with intercept and participant identifier as random effects, trials as a repeated measure, group status, spatial pattern separation distance and group status by spatial pattern separation distance interaction as fixed factors, and spatial pattern separation score as the outcome measure, controlling for age, gender (including gender by group status interaction), and years of education. Next, the test scores from each memory test (e.g., RAVLT 1–5, RAVLT-30, LM-IR, LM-DR, and ROCFT-R) were added sequentially to the LMM analyses to account for differences in memory performance. Receiver operating characteristic (ROC) analysis was utilised to evaluate the accuracy of the spatial pattern separation task in discriminating between groups. Areas under the ROC curves (AUC) with 95% confidence intervals (CI) are reported. The associations between regional brain measures, Aβ load, and spatial pattern separation performance were analysed using separate LMM with intercept and participant identifier as random effects, trials as a repeated measure, spatial pattern separation distance, volume of each selected brain region, number of Aβ-positive brain regions on amyloid PET imaging as fixed factors, and spatial pattern separation score as the outcome measure, controlling for age, gender, and years of education. The results are presented as unstandardized regression coefficients (β) with 95% confidence intervals (CI).

### Animal study

#### Animals

The study was conducted using 6-month-old male and female TgF344-AD (AD) rats and wild-type Fischer 344 (WT) rats. This age corresponds to an early preclinical window in TgF344-AD rats during which spatial memory deficits typically begin to emerge^52^. The animals (total *n* = 74) were assigned to four groups based on genotype and sex: AD males (*n* = 21), AD females (*n* = 17), WT males (*n* = 19) and WT females (*n* = 17). Rats were purchased from The Rat Resource and Research Center, University of Missouri, USA, under a valid MTA, and bred at the Institute of Physiology of the Czech Academy of Sciences. They were housed in pairs in transparent polycarbonate cages with wire mesh tops in a temperature-controlled colony room at a temperature of 22–23 °C under a 12:12 h light/dark cycle (lights on at 06:00). Food and water were available ad libitum. All behavioural testing was conducted during the light phase. In females, we did not monitor menopausal status, as 6-month-old Fischer-344 rats typically remain in regular oestrous cycles, with age-related changes such as cycle lengthening and irregularity occurring at later ages^53^. Also, the oestrous cycle was not staged because training spanned multiple consecutive days covering all stages of the regular 4-day cycle typical at this age, so any transient phase effects would be averaged across sessions. Moreover, daily vaginal cytology introduces handling-related stress and a sex-specific procedure, which can itself alter behaviour and endocrine state^54^. The experiments and housing conditions were approved by the Animal Care and Use Committee of the Institute of Physiology of the Czech Academy of Sciences, as well as by the Resort Professional Commission of the Czech Academy of Sciences for Approval of Projects of Experiments on Animals (51–2022-P). The experiments were conducted under veterinary supervision in compliance with Act No. 246/1992 Coll. and Decree No. 419/2012 Coll., which implement Directive 2010/63/EU of the European Parliament and the Council regarding the protection of animals used for scientific purposes. We adhered to the ARRIVE guidelines and maximised the application of the principles of the 3Rs.

#### Spatial pattern separation task in the morris water maze

**Fig. 2:**
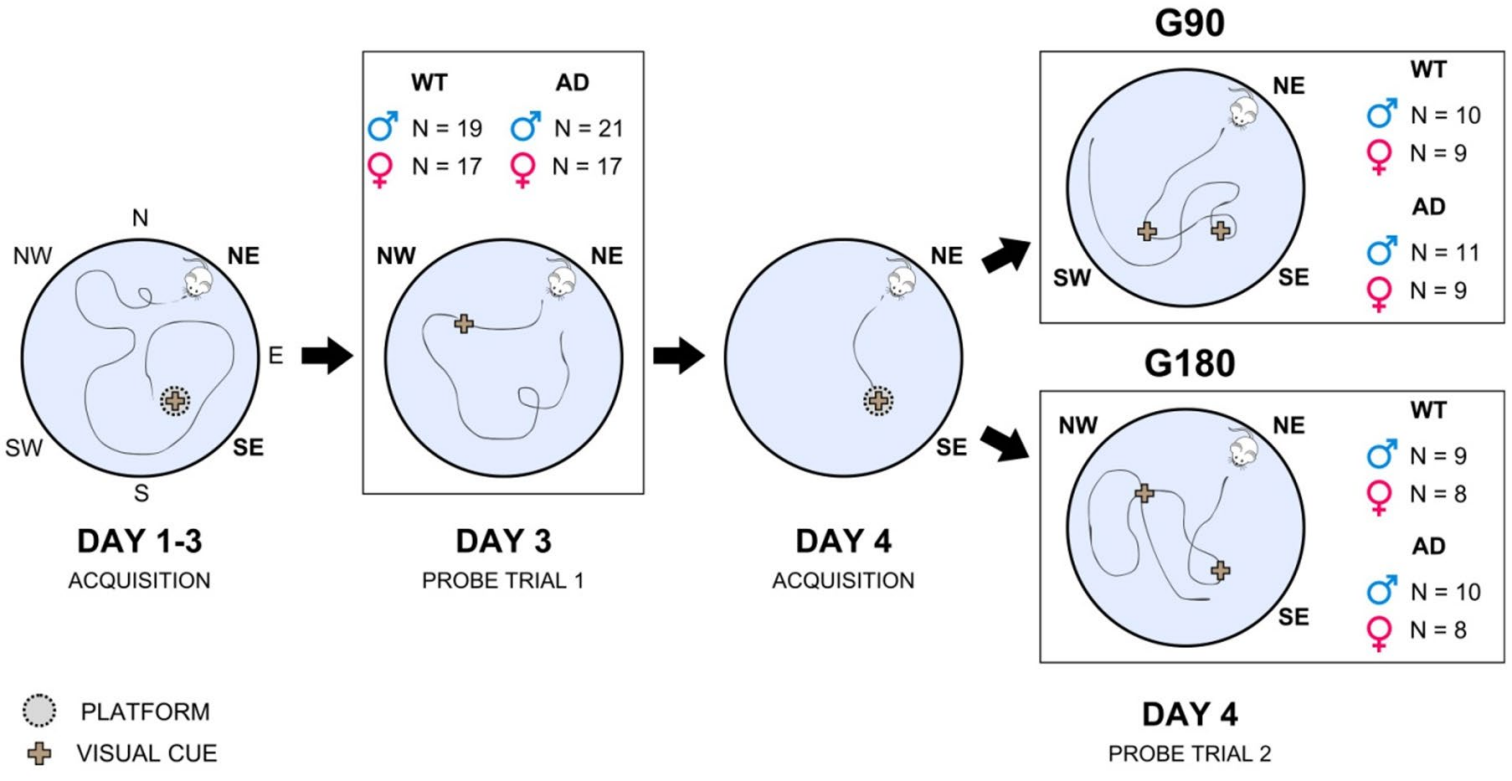
Experimental design of the Spatial Pattern Separation Task in rats. During Days 1–4 (the visible and hidden platform learning phases), the rodents learnt to locate a platform in a fixed location (in the SE quadrant) with a visual cue above it. On Day 3 (Probe Trial 1), the platform was removed and the movements of the rodents were tracked for a period of 60 seconds. On Day 4, immediately following the acquisition, the Probe Trial 2 was conducted, with the rodents being split into two groups: the G90 group (with visual cues in the SW and SE quadrants) and the G180 group (with visual cues in the NW and SE quadrants), in order to assess their adaptation to cue changes. N north; NE, north-east sector; E, east; SE, south-east sector; S, south; SW, south-west sector; NW, north-west sector; WT, wild-type rats; AD, transgenic TgF344-AD rats.

A modified version of the Morris Water Maze (MWM) task was used to assess spatial learning and the ability to distinguish between identical spatial visual cues in two different positions. Our modified MWM task focuses on spatial pattern separation and is specifically designed to assess fine discrimination rather than general acquisition. In this task, animals learn the location of a hidden platform and are then tested with two angular separations between competing distal cues. This manipulation targets interference control and complements the traditional MWM, which emphasises broad spatial learning. The maze consisted of a circular pool (1.8 m in diameter) filled with water maintained at 22 °C and made opaque using non-toxic black paint. The pool was surrounded by external distal cues, and a platform (10 cm diameter) was used for the animals‘ escape. The experiment was conducted over four consecutive days. Each animal completed eight training trials per day, with an additional probe trial conducted on days 3 and 4 following the eight training trials. The experimental design and groups are illustrated in Fig. 2.

##### Day 1 (Visible platform Training)

The platform was placed 1 cm above the water surface, and its location, as well as the animal’s starting position, were changed for each trial. A visual cue (two cardboard circles affixed perpendicularly to form a three-dimensional cross with a diameter of 18 cm) was placed above the platform to facilitate navigation.

##### Days 2–4 (Hidden platform Training)

The platform remained in a fixed location in the south-east (SE) sector, submerged 1 cm below the water’s surface. The visual cue remained visible 35 cm above the platform. Starting positions varied pseudorandomly across trials and days.

##### Day 3 (Probe Trial 1)

Following the eighth trial, a probe trial was conducted without the platform present. The visual cue was relocated to the north-west (NW) sector, after which the animals were observed for 60 seconds to assess their search strategy and their use of visual cues. The starting point was in the north-east (NE) sector.

##### Day 4 (Probe Trial 2)

Following the eighth trial, an additional probe trial was conducted, incorporating two visual cues. The visual cues were placed either in the south-west (SW) sector (90° from the original platform location) and the SE sector (platform location) for the Group 90° (G90) of animals, or in the NW sector (180° from the original platform location) and the SE sector (platform location) for the Group 180° (G180). The starting point was once again located at NE. The animals were randomly assigned to these groups, with consideration given to their sex and original cage location. The final distribution of animals was as follows: in the G90 group, female WT (*n* = 9), female AD (*n* = 9), male WT (*n* = 10), and male AD (*n* = 11); in the G180 group, female WT (*n* = 8), female AD (*n* = 8), male WT (*n* = 9), and male AD (*n* = 10). One AD female from the G180 group was excluded from the analysis due to missing data.

#### Data analysis

In the spatial pattern separation experiment in MWM, only the escape latency parameter was analysed during the acquisition phase (days 1–4). During the probe trials 1 and 2 (days 3 and 4), we analysed the time taken to reach the first platform and cue zone, the average distance from the platform and cue zone, and the total distance swum. For the purposes of analysis, the platform zone was defined as an area of the same size and location as the original platform. The cue zone was defined as an area of the same size located beneath the visual cue. It was essential to evaluate the relationship to the platform and cue zones separately, as we aimed to investigate how spatial orientation depends on spatial cues and how different distances of these cues might disrupt spatial navigation.

Statistical analyses were conducted using GLM in IBM SPSS Statistics 27. The level of statistical significance was set at *p* < 0.05. All data are presented as the median and interquartile range. Levene’s test was used to assess the homogeneity of variances for each dependent variable. All results were non-significant (*p* > 0.05), indicating comparable variances across groups. The Shapiro–Wilk test was used to evaluate normality, and variables with non-normal distributions were transformed using either a natural logarithm or Box–Cox transformation (λ = −0.13 to 2.74). Greenhouse–Geisser corrections were applied when the sphericity assumption was violated, as indicated by non-integer degrees of freedom.

To examine performance during the acquisition phase, two separate GLM analyses were conducted: one for the visible platform training on day 1, and another with repeated measures for the hidden platform training on days 2–4. In both models, sex and genotype (AD versus WT) were included as between-subjects factors.

For the probe trials, sex and genotype were included as between-subjects factors in Probes 1 and 2. Probe 2 also accounted for group differences based on the positions of visual cues. To evaluate average distance from the platform or cue during the 60-second trial, only the initial 15-second interval segmented into 5-second time bins was analysed, as this time window most accurately reflects goal-directed search behaviour^55^. In contrast, total distance travelled and time taken for the first crossing of the platform or cue zone were evaluated over the full 60-second trial. When significant main effects or interactions were detected, pairwise comparisons were conducted using the Holm–Bonferroni correction to examine the differences between the groups more closely.

## Results

### Results of the human study

**Fig. 3:**
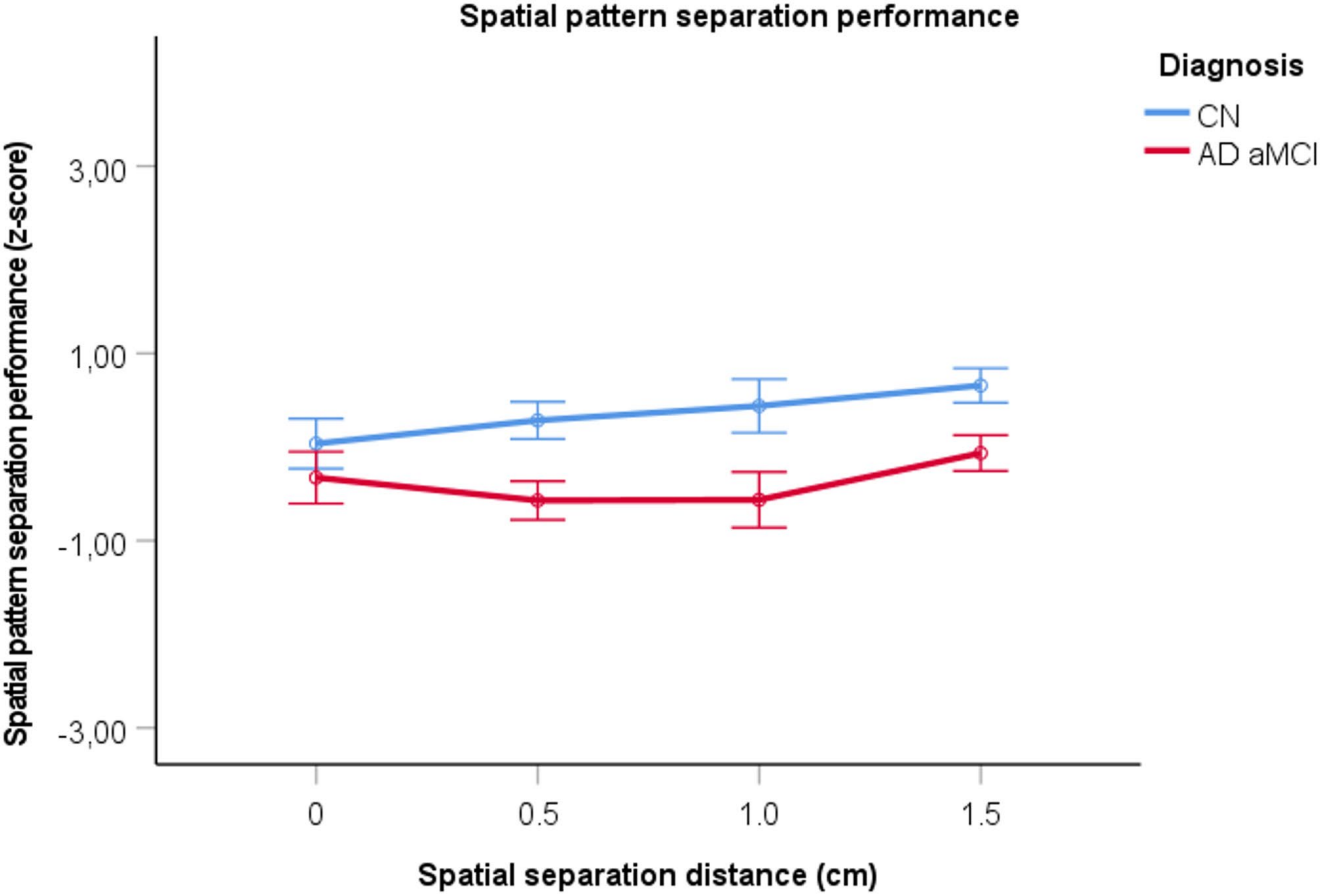
Spatial pattern separation performance in humans. Correct performance (z-score) for each spatial separation (mean ± 1 SE). **p < 0.01 and ***p < 0.001. CN, cognitively normal; AD aMCI, amnestic mild cognitive impairment with positive Alzheimer’s disease biomarkers.

Table 1 shows the demographic, cognitive, biomarker, MRI brain, and amyloid PET imaging characteristics. The AD aMCI group was older, had a lower proportion of women, performed worse on most cognitive tests, and had lower volumes of the hippocampus, entorhinal cortex and their subregions than the CN group. Figure 3 shows the differences in spatial pattern separation performance between the groups. On the Spatial Pattern Separation Task, the AD aMCI group performed worse than the CN group (F_(1, 303.139)_ = 9.578, *p* = 0.002, η^2^ = 0.086). There was a significant effect of spatial pattern separation distance (F_(1, 210.549)_ = 37.745, *p* < 0.001, η^2^ = 0.286), indicating that the greater the distance between the target and the foil circles, the better the performance. The effect of the interaction between spatial pattern separation distance and group status was not significant (F_(1, 210.549)_ = 0.389, *p* = 0.533, η^2^ = 0.003), indicating that the associations between spatial pattern separation distance and task performance did not differ between groups. The results also showed an effect of age (F_(1, 106.042)_ = 5.560, *p* = 0.020, η^2^ = 0.048), indicating that performance worsened with age, and an effect of gender (F_(1, 106.042)_ = 5.521, *p* = 0.021, η^2^ = 0.048), indicating that women performed worse than men. However, the effects of education (F_(1, 106.042)_ = 0.437, *p* = 0.510, η^2^ = 0.004) and the interaction between gender and group status (F_(1, 106.042)_ = 0.612, *p* = 0.436, η^2^ = 0.006) were not significant. The task discriminated between the AD aMCI and CN groups with an AUC of 0.70 (95% CI 0.66 to 0.75, *p* < 0.001). Controlling for memory test scores did not affect the findings of significant between-group differences, a significant effect of spatial pattern separation distance, or a nonsignificant interaction between spatial pattern separation distance and group status (see Supplementary Table 1).

**Table 1:** Demographic, cognitive, biomarker, MRI, and amyloid PET imaging characteristics.

	CN (*n* = 60)	AD aMCI (*n* = 56)	Total cohort (*n* = 116)	F/Χ^2^	*P*	Effect sizes
*Demographic characteristics*						
Age (years)	70.00 (5.12)	73.04 (6.73)	71.47 (6.12)	7.54	0.007	0.510
Women, n (%)	48 (80)	31 (55)	79 (68)	8.10	0.004	0.264
Education (years)	15.70 (2.15)	15.09 (3.20)	15.41 (2.71)	1.48	0.227	0.266
*Cognitive characteristics*						
GDS-15 (score)	0.90 (1.55)	2.24 (2.22)	1.54 (2.01)	9.80	0.002	0.083
BAI (score)	5.07 (4.91)	7.61 (8.01)	6.29 (6.69)	2.69	0.104	0.024
AVLT 1–5 (score)	55.27 (6.97)	34.14 (7.62)	45.66 (12.81)	175.86	< 0.001	0.626
AVLT 30 (score)	11.42 (2.24)	3.40 (3.32)	7.77 (4.87)	181.65	< 0.001	0.634
Logical memory IR (score)	18.20 (3.58)	9.59 (3.78)	14.04 (5.66)	116.43	< 0.001	0.512
Logical memory DR (score)	17.07 (3.88)	4.86 (5.12)	11.17 (7.60)	165.34	< 0.001	0.598
TMT A (seconds)	40.52 (11.16)	53.02 (28.24)	46.55 (22.02)	6.40	0.013	0.055
TMT B (seconds)	86.41 (28.41)	166.53 (88.01)	124.72 (75.52)	38.06	< 0.001	0.257
Phonemic VF (score)	48.35 (9.55)	42.09 (10.51)	45.33 (10.46)	6.47	0.012	0.055
ROCFT-C (score)	30.91 (3.13)	27.60 (4.10)	29.33 (3.98)	0.76	0.386	0.007
ROCFT-R (score)	18.23 (5.92)	8.10 (5.79)	13.38 (7.74)	73.16	< 0.001	0.399
DSF (score)	9.07 (2.03)	8.63 (1.89)	8.85 (1.97)	0.35	0.555	0.003
DSB (score)	6.58 (2.16)	5.66 (1.74)	6.14 (2.01)	3.83	0.053	0.033
CDT (score)	15.12 (1.37)	13.89 (1.93)	14.53 (1.77)	12.65	< 0.001	0.104
Semantic VF Animals (score)	27.00 (5.32)	18.55 (4.76)	22.92 (6.58)	60.01	< 0.001	0.351
BNT (score)	27.62 (3.86)	25.36 (3.35)	26.53 (3.78)	13.46	0.001	0.108
*Biomarker characteristics* ^*a*^						
CSF amyloid-β_1–42_ (pg/ml)	N/A	487.82 (128.00)	N/A	N/A	N/A	N/A
CSF p-tau_181_ (pg/ml)	N/A	110.20 (84.62)	N/A	N/A	N/A	N/A
CSF total tau (pg/ml)	N/A	539.30 (240.77)	N/A	N/A	N/A	N/A
*MRI characteristics* ^*b*^						
Hippocampus (volume, cm3)^c^	5.69 (0.59)	5.05 (0.83)	5.40 (0.78)	18.58	< 0.001	0.168
Anterior hippocampus (volume, cm3)^c^	3.13 (0.47)	2.86 (0.55)	3.01 (0.52)	9.91	0.002	0.097
Posterior hippocampus (volume, cm3)^c^	2.56 (0.28)	2.20 (0.39)	2.39 (0.38)	18,99	< 0.001	0.171
Entorhinal cortex (volume, cm3)^c^	2.14 (0.24)	1.91 (0.32)	2.03 (0.30)	20.75	< 0.001	0.186
Anterolateral entorhinal cortex (volume, cm3)^c^	1.40 (0.18)	1.25 (0.24)	1.33 (0.22)	15.04	< 0.001	0.142
Posteromedial entorhinal cortex (volume, cm3)^c^	0.74 (0.08)	0.66 (0.10)	0.70 (0.10)	25.03	< 0.001	0.216
Basal forebrain (volume, cm3)^c^	0.60 (0.11)	0.55 (0.09)	0.58 (0.11)	3.24	0.075	0.034
Basal forebrain Ch1-2 nuclei (volume, cm3)^c^	0.11 (0.02)	0.10 (0.02)	0.11 (0.02)	2.29	0.134	0.024
*Amyloid PET characteristics* ^*d*^						
Aβ-positive brain regions	N/A	6.33 (1,54)	N/A	N/A	N/A	N/A

Values are mean (SD) except for gender. *F*/Χ^2^ and *P* values refer to the main effect between CN and AD aMCI groups. Effect sizes were calculated as Cramér’s V for the χ^2^ test (gender), Cohen’s d for *t*-tests (other demographic characteristics), and partial eta-squared for general linear models (cognitive and MRI characteristics).

^a^Based on a sample with CSF data (*n* = 39).

^b^Based on a sample with complete MRI data (*n* = 96) with CN (*n* = 52) and AD aMCI (*n* = 44).

^c^Normalized to estimated total intracranial volume.

^d^Based on a sample with amyloid PET data (*n* = 30).

CN, cognitively normal; AD aMCI, amnestic mild cognitive impairment with positive Alzheimer’s disease biomarkers; APOE, Apolipoprotein E; GDS-15, Geriatric Depression Scale 15-item version; BAI, Beck Anxiety Inventory; AVLT, Rey Auditory Verbal Learning Test; AVLT 1–5, trials 1 to 5 total; AVLT 30, delayed word recall after 30 minutes; IR, Immediate Recall; DR, Delayed recall; TMT A and B, Trail Making Tests A and B; Phonemic VF, Phonemic Verbal Fluency (Czech version with letters N, K and P); ROCFT-C, Rey-Osterrieth Complex Figure Test – the Copy condition; ROCFT-R, Rey-Osterrieth Complex Figure Test – the Recall condition after 3 minutes; DSF, Digit Span Forward total score; DSB, Digit Span Backward total score; CDT, Clock Drawing Test – Cohen’s scoring; Semantic VF, Semantic Verbal Fluency; BNT, Boston Naming Test (30-item version); CSF, cerebrospinal fluid; MRI, magnetic resonance imaging; amyloid PET, amyloid positron emission tomography; Aβ, amyloid beta.

The analyses of associations between regional brain measures and spatial pattern separation performance demonstrated that a smaller volume of the posterior hippocampus was associated with worse performance (β = 0.833, *p* = 0.023, 95% CI 0.119 to 1.547). The associations between volumes of the anterior hippocampus (β = 0.254, *p* = 0.384, 95% CI −0.324 to 0.833), total hippocampal volume (β = 0.341, *p* = 0.077, 95% CI −0.038 to 0.719), and spatial pattern separation performance were not significant. A smaller volume of the entorhinal cortex was associated with worse performance (β = 1.000, *p* = 0.022, 95% CI 0.149 to 1.850). This association was driven by the association between volume of the posteromedial entorhinal cortex and spatial pattern separation performance (β = 3.422, *p* = 0.008, 95% CI 0.923 to 5.920), as the association between volume of the anterolateral entorhinal cortex and task performance was not significant (β = 1.148, *p* = 0.056, 95% CI −0.028 to 2.324). A smaller volume of the basal forebrain Ch1-2 nuclei was associated with worse performance (β = 18.767, *p* = 0.007, 95% CI 5.235 to 32.298). The association between total basal forebrain volume and spatial pattern separation performance was not significant (β = 4.681, *p* = 0.283, 95% CI −3.936 to 13.299). The analyses of associations between semi-quantitative measures of amyloid PET images and spatial pattern separation performance did not reveal any significant associations between the number of Aβ-positive brain regions and task performance (β = 0.277, *p* = 0.088, 95% CI −0.450 to 0.600).

### Results of the animal study

#### Acquisition days 1–4 (Visible and Hidden platform)

Analysis of escape latency during the acquisition phase using a univariate GLM revealed distinct patterns in the visible and hidden platform phases. On day 1 (visible platform), no effects of genotype, sex, or their interactions were observed. On days 2–4 (hidden platform), significant effects of day and day-by-sex interaction were observed, again with no influence of genotype, sex, or their interactions. The subsequent section provides a detailed breakdown of the escape latency data for the hidden platform phase.

On days 2–4 (hidden platform), the analysis revealed significant changes in escape latency over time, with performance improving across days (F_(2, 140)_ = 136.88, *p* < 0.001, η^2^ = 0.662). To explore these changes in more detail, repeated contrasts were conducted across the three testing days, correcting for multiple comparisons. Repeated contrasts revealed significant differences in escape latency between days 2 and 3, and between days 3 and 4 (both *p* < 0.001), reflecting progressive improvement across days. A significant interaction between day and sex (F_(2, 140)_ = 3.371, *p* < 0.026, η^2^ = 0.051) was also observed. However, post-hoc tests did not reveal any significant sex differences within individual days.

#### Probe trial 1 (day 3)

A repeated-measures GLM was conducted to assess the effect of time, sex, genotype, and their interactions on the average distance from the platform and cue. A multivariate GLM examined the effects of sex, genotype, and their interaction on the time taken to first entry into the platform and cue zones, as well as on the total distance swum. Significant effects of time were found for the average distance from both the platform and the cue. The analyses also revealed a main effect of sex, with females having a greater average distance from the platform than males. Additionally, the multivariate GLM revealed significant effects of sex on the time taken to first platform entry and total distance swum, as well as of genotype on total distance swum. A significant sex-by-genotype interaction was also observed for total distance swum. Post hoc comparisons revealed that AD females swam greater total distances than WT females. Details of these effects are reported below.

**Fig. 4:**
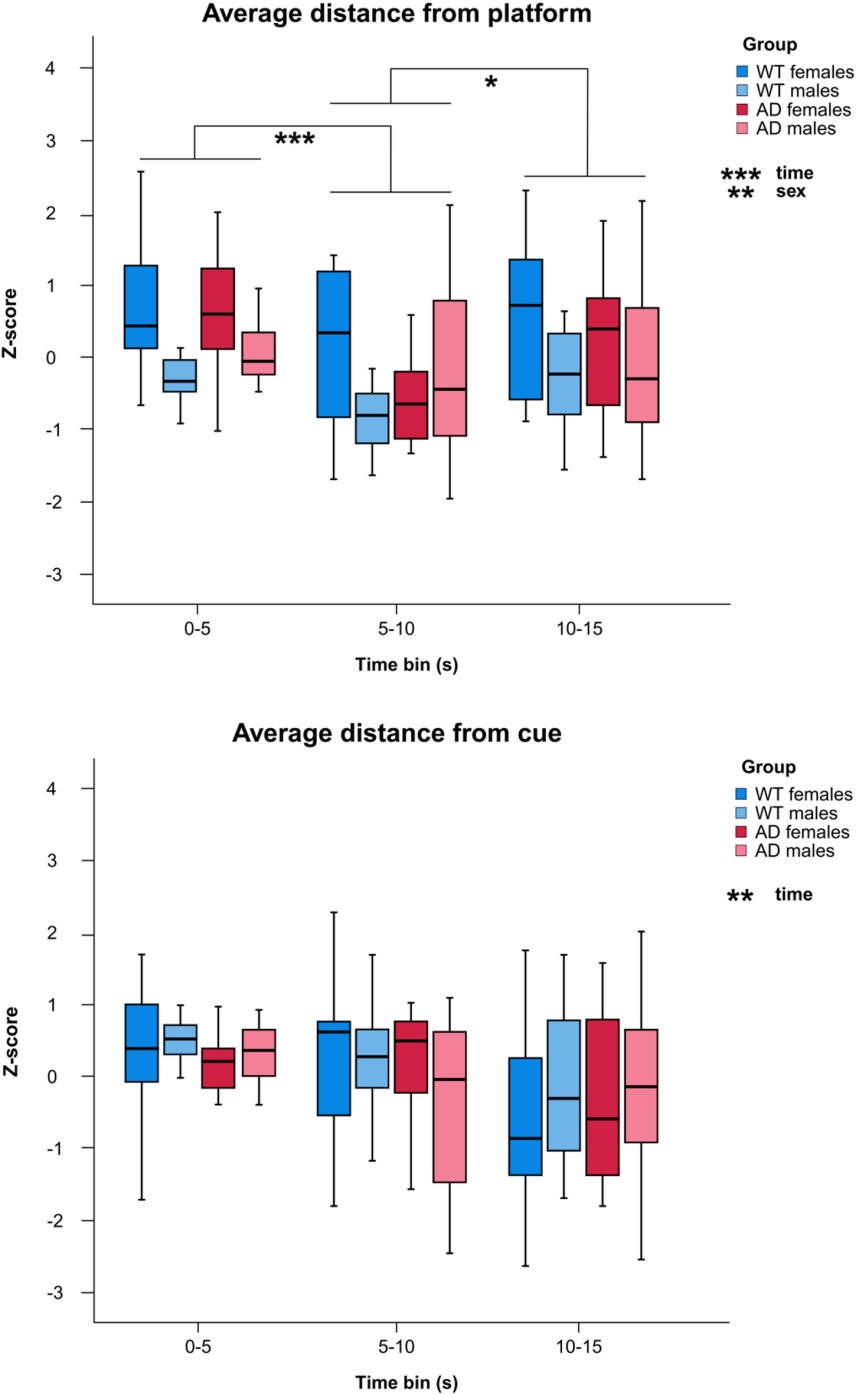
Spatial memory performance in rats. Average distance from the platform and cue (z-score) for 0–5 second, 5–10 second, and 10–15 second time bins in Probe Trial 1 (Day 3). Boxplots represent the median and interquartile range. * *p* < 0.05, ** *p* < 0.01, *** *p* < 0.001. WT, wild-type rats; AD, transgenic TgF344-AD rats.

A significant main effect of time was observed in the average distance from the platform (F_(1.829, 128.052)_ = 11.882, *p* = 0.001, η^2^ = 0.145), indicating a significant change in the average distance from the platform over time. Repeated contrasts revealed significant changes in performance between the 0–5 second and 5–10 second time bins (*p* < 0.001), reflecting a decrease in the average distance from the platform, and between the 5–10 second and 10–15 second time bins (*p* = 0.014), reflecting an increase in distance. In addition, a significant main effect of sex was found (F_(1, 70)_ = 10.524, *p* = 0.002, η^2^ = 0.131), with females having a greater average distance from the platform than males. No effects of genotype or interactions between factors were observed. A significant main effect of time was observed in the average distance from the cue (F_(2, 140)_ = 4.905, *p* = 0.009, η^2^ = 0.843). However, the repeated contrasts analysis revealed no significant changes over the three time points. The results are shown in Fig. 4.

A significant main effect of sex was observed on the time taken to first entry into the platform zone (F_(1, 69)_ = 16.877, *p* < 0.001, η^2^ = 0.197), with males reaching the platform zone earlier than females. A significant main effect of genotype was observed for total distance swum (F_(1, 69)_ = 13.321, *p* < 0.001, η^2^ = 0.162), with AD rats swimming longer distances than WT rats. A significant main effect of sex was also observed for total distance swum (F_(1, 69)_ = 7.620, *p* = 0.007, η^2^ = 0.099), with males swimming shorter distances than females. Additionally, a significant interaction between sex and genotype was found for total distance swum (F_(1, 69)_ = 5.382, *p* = 0.023, η^2^ = 0.072). To further explore this effect, separate univariate GLM analyses were conducted for males and females, with genotype as the fixed factor. This follow-up analysis revealed a significant genotype effect only in females (F_(1, 31)_ = 17.321, *p* < 0.001, η^2^ = 0.358), indicating that AD females swam a greater total distance than WT females (Supplementary Fig. 1).

#### Probe trial 2 (day 4)

A repeated-measures GLM was conducted to assess the effect of time, sex, genotype, and their interactions on the average distance from the platform and cue zones across three time points in the G90 and G180 groups on probe day 4. A multivariate GLM examined the effects of sex, genotype, and their interaction on the time taken to first entry into the platform and cue zones. In summary, the analyses revealed a significant main effect of time on average distance from the platform in the G90 group, with post hoc contrasts confirming differences between 0 and 5 second and 5–10 second time bins. Additionally, a significant time-by-genotype interaction was observed, with AD animals displaying a significantly higher average distance from the platform than WT animals within the 0–5 second interval. In the G180 group, only a significant main effect of time on average distance from the platform was found, supported by post hoc contrasts showing a change between the 0–5 second and 5–10 second time bins. A significant main effect of time and sex was detected for average distance from the cue in the G90 group, but not in the G180 group. A multivariate GLM revealed that males in the G90 group reached the platform zone significantly faster than females. Regardless of cue condition, AD animals swam longer distances than WT controls. Details of these effects are reported below.

**Fig. 5:**
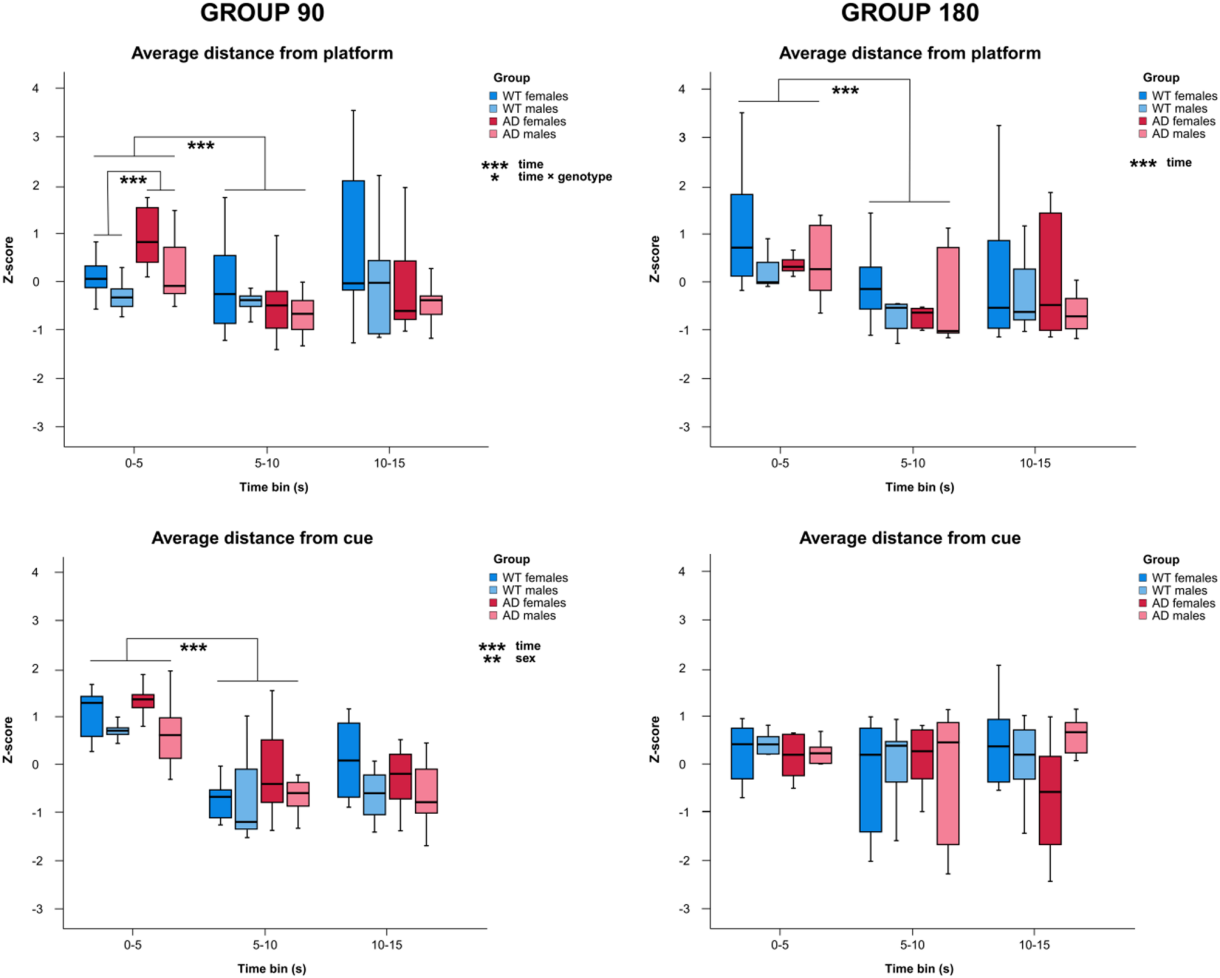
Spatial pattern separation performance in rats. Average distance from the platform and cue (z-score) for 0–5 second, 5–10 second, and 10–15 second time bins for the G90 and G180 groups in Probe Trial 2 (Day 4). Boxplots represent the median and interquartile range. * *p* < 0.05, ** *p* < 0.01, *** *p* < 0.001. WT, wild-type rats; AD, transgenic TgF344-AD rats.

**Fig. 6:**
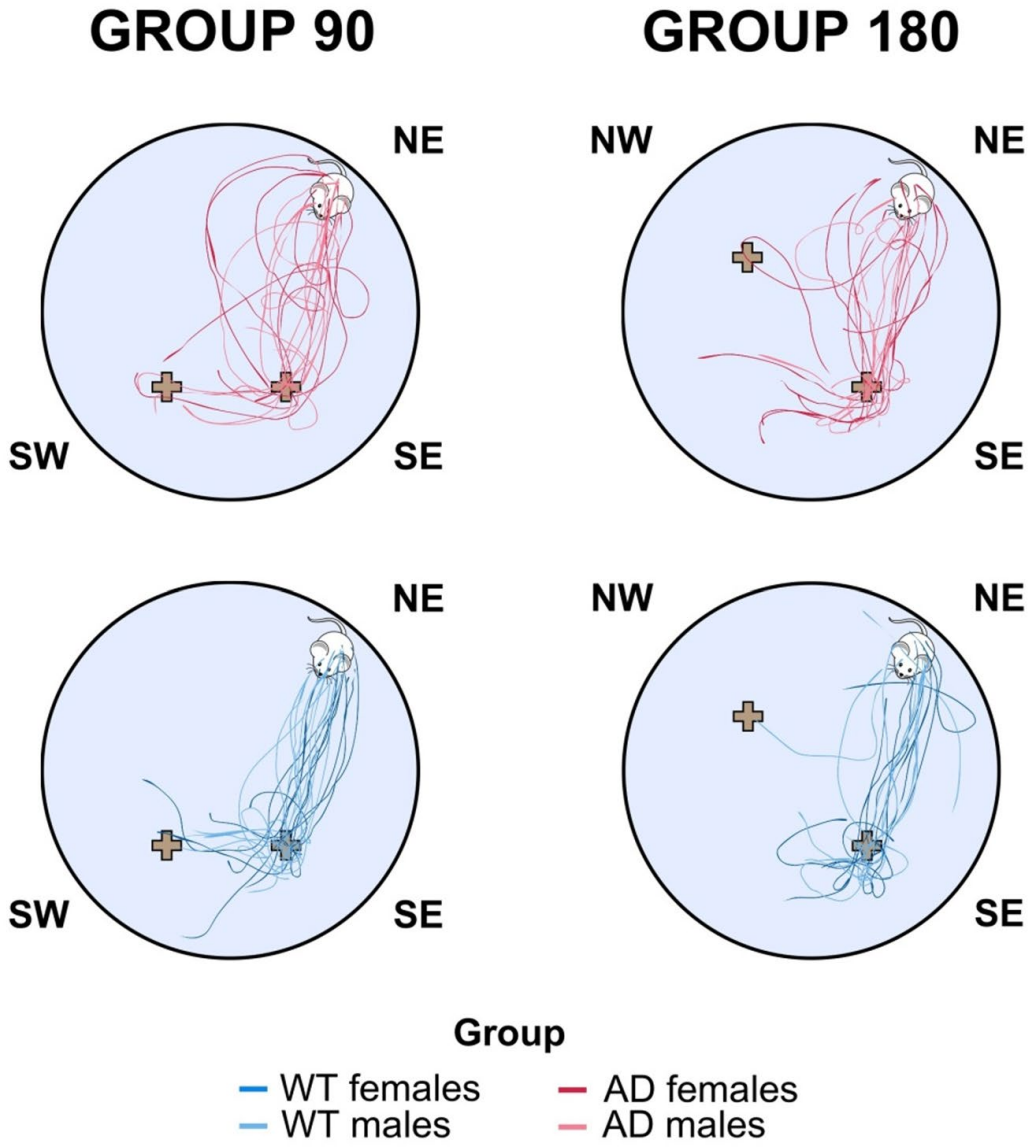
Swimming trajectories in the Spatial Pattern Separation Task in rats. The swimming trajectories in the 0–5 second time bin for the G90 and G180 groups in Probe Trial 2 (Day 4). NE, north-east sector; SW, south-west sector; SE, south-east sector; NW, north-west sector; WT, wild-type rats; AD, transgenic TgF344-AD rats.

The main findings of this study are based on the analyses of the average distances from the platform and cue zones conducted in the G90 and G180 groups on probe day 4. The results are shown in Fig. 5. Analysis of the G90 group revealed a significant main effect of time on the average distance from the platform (F_(2, 74)_ = 6.97, *p* = 0.002 η^2^ = 0.211). Repeated contrasts revealed significant differences between the 0–5 second and 5–10 second intervals (*p* < 0.001), indicating a reduction in the average distance from the platform. No significant main effect of sex, genotype or sex-by-genotype interaction was observed. However, a significant time-by-genotype interaction was revealed (F_(2, 74)_ = 4.496, *p* = 0.014 η^2^ = 0.108). Multivariate GLM analysis of each time bin showed that AD rats had a significantly greater average distance from the platform than WT rats in the 0–5 second time bin (F_(1, 40)_ = 12.051, *p* = 0.001, η^2^ = 0.236). This discriminated between the AD and WT groups with an AUC of 0.785 (95% CI 0.645 to 0.924, *p* = 0.002). The trajectories of rats in the 0–5 second time bin on probe day 4 are shown in Fig. 6. In the G180 group, the statistical analysis revealed only a significant main effect of time (F_(2, 56)_ = 13.344, *p* < 0.001, η^2^ = 0.323). Repeated contrasts indicated a significant difference between the 0–5 second and 5–10 second time bins (*p* < 0.001), indicating a decrease in the average distance from the platform. No significant effects of genotype, sex or interactions between factors were detected in the G180 group.

A repeated-measures GLM revealed a significant main effect of time on the average distance from the cue in the G90 group (F_(2,74)_ = 41.356, *p* < 0.001, η^2^ = 0.955). Follow-up repeated contrasts revealed a significant difference between the 0–5 second and 5–10 second time bins (*p* < 0.001), indicating a reduction in the distance to the cue. There was also a significant main effect of sex (F_(1, 37)_ = 9.479, *p* = 0.004, η^2^ = 0.196), with males maintaining a shorter average distance to the cue than females. No effects of genotype or interactions between factors were observed in the G90 group. In the G180 group, no significant main effects of any factor or interactions between factors were observed.

A multivariate GLM was used to assess the effects of sex, genotype, and their interaction on the time taken to first entry into the platform and cue zones on day 4 in the G90 and G180 groups. In the G90 group, a significant main effect of sex was found for the time taken to first entry into the platform zone (F_(1, 37)_ = 5.033, *p* = 0.031, η^2^ = 0.120), with males reaching the zone earlier than females. No effects of genotype or sex-by-genotype interactions were observed. No significant main effects of sex or genotype, nor any interaction effects between factors, were observed for the time taken to first cue zone entry in the G90 group. No significant main effects of sex or genotype were found for the time taken to first entry into the platform and cue zone in the G180 group, and no interaction effect was detected. The total distance was analysed using a univariate GLM, without separating it by cue condition. Only a significant main effect of genotype emerged (F_(1, 69)_ = 7.709, *p* = 0.007, η^2^ = 0.100), with AD animals swimming longer distances than WT controls (Supplementary Fig. 1).

## Discussion

The present translational study utilised human and novel animal versions of the Spatial Pattern Separation Task in biomarker-defined participants with early AD and in a rodent model of the disease. The study yielded the following key findings: Firstly, participants with AD aMCI performed less accurately than CN participants, with a significant effect of spatial pattern separation distance observed in both groups. Secondly, these results remained essentially unchanged after adjusting for memory performance. Thirdly, in the probe trial with a 90° pattern separation design, six-month-old TgF344-AD rats performed worse than WT rats. However, no differences were observed in the probe trial with a 180° design. Fourthly, TgF344-AD and WT rats performed similarly in a probe trial that assessed spatial memory with no pattern separation demands. Thus, our findings extend the current knowledge by demonstrating that spatial pattern separation deficits are present in the early stages of AD in both humans and rodent models, and that these deficits are not attributable to memory impairment. This makes spatial pattern separation testing a promising approach for translational AD research.

In the human part of the study, the Spatial Pattern Separation Task demonstrated a good discriminatory power in identifying participants with early AD. These results corroborate the earlier findings, showing the potential of the task to differentiate individuals with biomarker-defined early AD from CN older adults^21,26^. Furthermore, our current results showed that the differences between the groups remained essentially unchanged when verbal and non-verbal memory was taken into account in the analyses. Therefore, poorer performance in the early stages of AD is unlikely to be due to general memory deficits, but rather reflects less efficient spatial pattern separation. Our results also showed that task performance declined as the spatial distance decreased across groups, which further confirms that performance reflects spatial pattern separation processes^21^.

In the human part of the study, we measured volumes of the hippocampal and entorhinal cortex subregions, which are crucial for pattern separation, to evaluate structural brain changes that may underlie spatial pattern separation deficits. We found that smaller volumes of the posterior hippocampus, including the body and tail, and the posteromedial entorhinal cortex were associated with less accurate spatial pattern separation performance. In contrast, the volumes of the anterior hippocampus (i.e., the head) and anterolateral entorhinal cortex were not associated with this performance. These findings align with our previous study, which demonstrated associations between the volumes of the posterior hippocampus and posteromedial entorhinal cortex and spatial pattern separation impairment^21^. They also extend previous research showing the associations between functional changes in the posterior hippocampus^56^ and posteromedial entorhinal cortex^57^ and performance in spatial discrimination tasks. We also measured the volume of the basal forebrain Ch1-2 nuclei, which directly project to the hippocampus and entorhinal cortex, to evaluate the link between their structural changes and spatial pattern separation deficits. Lower volume of the basal forebrain Ch1-2 nuclei, unlike the total basal forebrain volume, was associated with less accurate spatial pattern separation performance. These findings are consistent with our previous studies^21,23^, as well as animal research, which has reported specific cholinergic projections from the basal forebrain Ch1-2 nuclei to the hippocampus and entorhinal cortex^50,58^, and impaired spatial pattern separation resulting from the disruption of basal forebrain cholinergic projections to the hippocampus^20^. Finally, we measured the accumulation of Aβ and examined its association with spatial pattern separation impairment. Our findings did not show any associations between Aβ load and spatial pattern separation performance. Previous studies have reported that spatial discrimination deficits are associated with higher cortical Aβ accumulation^59,60^. However, it is worth noting that there were methodological differences between our study and the previous studies. In our study, we visually assessed the number of Aβ-positive brain regions, while the previous studies measured Aβ deposition quantitatively. The plausible explanation for our finding is that the overall degree of Aβ burden may be more closely associated with cognitive impairment than the Aβ distribution, as Aβ accumulates throughout the entire neocortex in the early stage of AD, in Thal stage 1^61^. More research is required to investigate the associations between visually and quantitatively measured Aβ pathology and spatial pattern separation performance in individuals with early AD.

The hippocampus plays a key role in pattern separation processes, which depend on information received from the entorhinal cortex via the perforant pathway^12^. The entorhinal cortex and the hippocampus are among the first cortical regions to exhibit tau pathology in Braak stages I–II and II–III, respectively^9^. Aβ pathology facilitates the spread of tau pathology^62^ and induces abnormal neural activity in the medial temporal lobe^63^. Therefore, deficits in spatial pattern separation in the early stages of AD can be attributed to tau and Aβ pathologies. Indeed, previous studies of cognitively normal older adults have shown that greater accumulation of tau in the medial temporal lobe and of Aβ in the neocortex is associated with less efficient pattern separation^59,60^. This accumulation has been linked to abnormal neuronal activity in the medial temporal lobe and dysfunction of the entorhinal–hippocampal circuit, leading to pattern separation deficits^64,65^. Another possible cause of disruption to spatial pattern separation processes can be AD-related dysfunction of the subcortical nuclei, primarily the basal forebrain and locus coeruleus. Pattern separation processes are modulated by acetylcholine^12^, the major source of which to the hippocampus and entorhinal cortex is the basal forebrain^58^. The basal forebrain accumulates tau pathology very early in the course of AD (at Braak stages I-II^16^ or even stage 0^18,58^), and its degeneration precedes and predicts EC pathology and neurodegeneration in preclinical AD^18,66^. It has been demonstrated that lesions to the basal forebrain cholinergic projections are associated with less effective spatial pattern separation^20^. Disruption of cholinergic projections results in low levels of this neurotransmitter in the hippocampus and entorhinal cortex, which decreases discrimination ability and biases hippocampal computation processes away from pattern separation^14^. In accordance with these findings, recent studies have demonstrated an association between neurodegeneration of the basal forebrain, especially Ch1-2 nuclei, and impaired spatial pattern separation^21,23^ in individuals with AD, where this association was fully mediated by neurodegeneration of the posterior hippocampus and posteromedial entorhinal cortex^23^. The locus coeruleus is the primary source of noradrenaline for the brain, and previous research has shown that it is the first region to accumulate hyperphosphorylated tau protein (corresponding to Braak stage 0), preceding the emergence of tau pathology in the medial entorhinal cortex^67,68^. The noradrenergic projections from the locus coeruleus preferentially terminate in the dentate gyrus of the hippocampus^69^, which plays a key role in pattern separation computations^10^. Moreover, activation of the locus coeruleus increases the synaptic strength of the perforant pathway, a critical input from the entorhinal cortex regulating dentate gyrus activity^70^. Previous studies have shown that noradrenergic activation enhances the ability of the dentate gyrus to discriminate among similar stimuli, thereby facilitating pattern separation^22^. Additionally, a recent study showed that activation of the locus coeruleus predicts memory separation and enhances pattern separation in the dentate gyrus^15^. Consequently, locus coeruleus-associated noradrenergic dysfunction could contribute to spatial pattern separation deficits in early AD.

In the animal experiment, the Spatial Pattern Separation Task, utilising a modified version of the MWM task, demonstrated good discriminatory capability in differentiating between six-month-old TgF344-AD and WT rats. Specifically, in the 90° pattern separation probe trial, the TgF344-AD rats exhibited a higher average distance from the hidden platform than their WT littermates in the 0–5 second time bin, but not in the 180° pattern separation probe trial or the probe trial with no pattern separation demands. The 90° separation represented a more challenging condition for TgF344-AD rats compared to the 180° separation. The AD animals exhibited greater early misorientation and less precise trajectories toward the platform with the smaller angular separation of 90°, resulting in a higher average distance to the platform at trial onset. In contrast, the larger angular separation of 180° reduced cue interference, resulting in performance that was more similar to WT rats. These findings are consistent with those of previous studies on mice, which showed that the 5xFAD^71^ and Tg2576^72,73^ mouse models of early-stage AD exhibit decreased accuracy in spatial pattern separation performance compared to WT mice. The deficits in spatial pattern separation performance in mouse models of AD have been predominantly linked to hippocampal dysfunction. Specifically, they have been associated with overactivation of the dentate gyrus^73^, hyperexcitability of granule cells in the dentate gyrus^74^, and degeneration of hippocampal cholinergic synapses^72^. Previous studies have not addressed spatial pattern separation performance directly in rat models of AD. However, research has demonstrated that rats with lesions to the hippocampus^75,76^ and the septo-hippocampal cholinergic neurons of the basal forebrain^20^, which are among the first to be affected by AD pathophysiological changes^9,18^, show deficits in discriminating between similar spatial locations in a dryland version of the MWM and on a touchscreen, especially when the locations are close together, indicating impaired spatial pattern separation performance.

The present study also found that six-month-old TgF344-AD rats performed similarly to WT rats in the hidden platform training trials and the probe trial on day three, demonstrating comparable spatial learning and memory abilities. These results are consistent with previous research showing that spatial learning and memory deficits are not observed in the TgF344-AD rat model of AD in the MWM task until 7–8 months of age, with these deficits becoming significantly more pronounced at 10–11 months of age, as indicated by longer escape latencies and greater search distances^77^. However, this previous research has also shown that seven- to eight-month-old TgF344-AD rats exhibit different swimming patterns compared to WT rats in the MWM task, with less direct swimming trajectories^77^. In the present study, significant differences were identified between six-month-old TgF344-AD and WT rats during the first five seconds of the 90° probe trial. This finding suggests that the spatial pattern separation deficits in TgF344-AD rats may partly reflect early deviations in search precision that occur before broader spatial memory deficits emerge. At the same age of 6 months in the TgF344-AD rats, where we observed a deficit in spatial pattern separation, previous studies reported reversal learning deficits in spatial tasks^24,78^. These behaviours depend on partially overlapping systems, with spatial pattern separation relying primarily on computations in the dentate gyrus and CA3 hippocampal regions, and reversal learning engaging broader prefrontal, cingulate, and striatal networks in interaction with the hippocampus^10,79^. A plausible common contributor is an early disruption of the noradrenergic system associated with the locus coeruleus. Converging evidence indicates very early, Braak I-II-like abnormalities in the locus coeruleus in 6-month-old TgF344-AD rats, including hyperphosphorylated tau pretangle accumulation and altered firing, leading to progressive loss of noradrenergic innervation in the medial entorhinal cortex and dentate gyrus^78,80,81^, and restoration of reversal learning deficits after chemogenetic activation of the locus coeruleus in 16-month-old TgF344-AD rats^78^, supporting a potential mechanistic link between early AD-related neuropathological changes and hippocampus-dependent memory discrimination.

It is important to note that the present study identified sex differences in spatial memory and spatial pattern separation. Female rats performed less accurately than male rats in the probe trial on day three and in the 90° pattern separation probe trial, as measured by a longer time to first entry into the platform zone. The present findings are consistent with the results of earlier research on sex differences, which have demonstrated that male rats outperform female rats in spatial memory performance in the MWM task^82,83^ and in spatial pattern separation^84^. In the latter, male rats exhibited greater neurogenesis in response to pattern separation training^84^. The present results demonstrated a similar adverse effect of AD pathology on spatial pattern separation performance in male and female rats, as evidenced by a negative interaction between sex and genotype in the average distance from the platform in the 90° pattern separation probe trial. Although we did not stage the oestrous cycle, a previous study^85^ has shown that landmark-based spatial learning in the MWM is not modulated by oestrous phase, suggesting that hormonal fluctuations across the cycle do not significantly affect spatial memory performance in female rats. Similar findings were obtained in the human part of the study, in which postmenopausal female participants performed worse than their male counterparts, with no statistically significant interaction observed between gender and group status. While these analogous findings in rats and humans are interesting and suggest sex-based differences in spatial pattern separation with limited susceptibility to AD pathology, their interpretation is not straightforward due to the fact that the female participants were postmenopausal and the female rats were hormonally active. Therefore, future studies involving female rats and human participants with similar hormonal profiles are needed to show whether spatial pattern separation is susceptible to AD pathophysiology, regardless of sex, which could emphasise the value of using spatial pattern separation tasks in translational research.

In the present study, the Spatial Pattern Separation Task was utilised, with modifications made to ensure its applicability to both rats and humans. A modified version of the MWM task was used in the animal experiment to examine spatial pattern separation. The methodological approach adopted was informed by prior research, which demonstrated that a task based on spontaneous spatial exploration exhibited superior efficacy in identifying mild spatial pattern separation deficits in the 5xFAD mouse model of early-stage AD, when compared with a touchscreen-based method^71^. In the human experiment, a validated spatial discrimination task with four separation distances was employed. The rationale underlying the selection of this particular task is based on the findings of previous research, which demonstrated its reliability in identifying the early stages of AD^21,26^. The present results demonstrated that the Spatial Pattern Separation Task has comparable discrimination capabilities in rats and humans with respect to identifying early-stage AD. Moreover, a similar spatial pattern separation property of the task was observed in both participants with AD and rat models, with performance declining as the distance between locations decreased.

Although we designed our experiment to be as similar as possible for rats and humans, there were still some notable differences, which can be considered limitations of the study. In contrast to the human part of the study, which utilised four separation distances, the animal experiment employed only two distances between the cue and the platform, 90° and 180°, corresponding to the adjacent and opposite quadrants, respectively. This difference in task design limits the quantitative comparability of our translational results. Unlike in the human study, where all participants underwent testing at all spatial separation distances, the rodents in the animal experiment were randomised into 90° and 180° spatial pattern separation conditions. This may limit the generalisability of the findings and reduce the statistical power to detect significant differences between the TgF344-AD and WT rats. A design incorporating additional separation distances would be valuable for future studies to further explore the impact of varying cue separations on performance across different experimental conditions. Furthermore, since a rodent model of sporadic AD is not available, we used a validated familial AD model^24^. However, this model may not accurately replicate the localisation or extent of the pathophysiological changes observed in people with sporadic AD^86,87^. A further limitation of the study is the absence of neuropathological data in the rodent model and the lack of quantitative measures of Aβ load in humans.

To strengthen the translational relevance of our findings, future studies should include physiological and molecular measures that parallel those assessed in early AD in humans. We selected the TgF344-AD model because it effectively replicates both early Aβ and tau pathology, along with glial activation, making it a promising translational model. Importantly, these changes are already detectable in the preclinical stage^24,88^ and correspond to biomarkers commonly evaluated in clinical cohorts. Moreover, several early circuit-level abnormalities in TgF344-AD rats are closely related to mechanisms involved in spatial pattern separation. By six months, dentate gyrus granule cells exhibit pronounced hyperexcitability and enhanced β-adrenergic-dependent long-term potentiation^89,90^, there is a functional reduction of cholinergic signalling despite increased synaptic density^91^, and a decline in locus coeruleus noradrenergic projections to the hippocampus^89^. Also, broader neurochemical, synaptic and glial expression changes become apparent with age^92^. Incorporating these physiological and molecular outcomes in future experiments would allow for a more mechanistically grounded interpretation of the behavioural spatial pattern separation phenotype within a translational framework.

In view of the considerable increase in the number of people affected by AD, there is an urgent need for non-invasive, cost-effective tools that can facilitate its early detection. This is a critical step towards the effective use of new disease-modifying therapies^2^. Spatial pattern separation assessment has emerged as a promising marker for the early detection of cognitive deficits associated with AD in humans^21^ and rodent models^71^. The present study demonstrated that a spatial pattern separation task with a similar design yielded comparable results when administered to both species, thereby enabling direct comparisons to be made between the cognitive performance of rats and humans. These findings could pave the way for spatial pattern separation tasks to be used to directly compare the effects of developing disease-modifying and symptomatic drugs in the preclinical and clinical phases of AD drug trials. The utilisation of these tasks has the potential to mitigate the observed discrepancies in cognitive outcomes between these phases^93^.

## Conclusions

The present study employed comparable animal and human protocols, yielding several significant findings that further advance our understanding of spatial pattern separation deficits in AD. Specifically, spatial pattern separation is impaired in the early stages of AD beyond general memory impairment, and its testing reliably discriminates between participants with early aMCI and CN participants. Importantly, analogous results were obtained in the animal experiment, as evidenced by impaired spatial pattern separation alongside unaffected spatial memory in six-month-old TgF344-AD rats compared to WT rats. These results provide support for the hypothesis that spatial pattern separation tasks have strong translational potential, opening new avenues for their use as outcome measures of cognitive functions in AD drug research.

## Supplementary Information

Below is the link to the electronic supplementary material.

Supplementary Material 1

## Data Availability

All primary data from this study are detailed within the article. Any additional information and dataset required to reanalyse the data reported in this paper are available from the corresponding authors upon request.
