# Supplementary material for: Spatial pattern separation deficits in early Alzheimer’s disease are comparable in humans and animal models
Source: Sci Rep. 2026 Jan 22;16:6020. doi: 10.1038/s41598-026-36266-y (PMC12902105; doi:10.1038/s41598-026-36266-y)
Supplement: Supplementary file 1 — Supplementary Material 1 [file 41598_2026_36266_MOESM1_ESM.docx]

**Supplementary Figure 1** Total distance swam in rats. The total distance swum (z-score) in Probe Trials 1 and 2. Boxplots represent the median and interquartile range. * p < 0.05, ** p < 0.01, *** p < 0.001. WT, wild-type rats; AD, transgenic TgF344-AD rats.

**
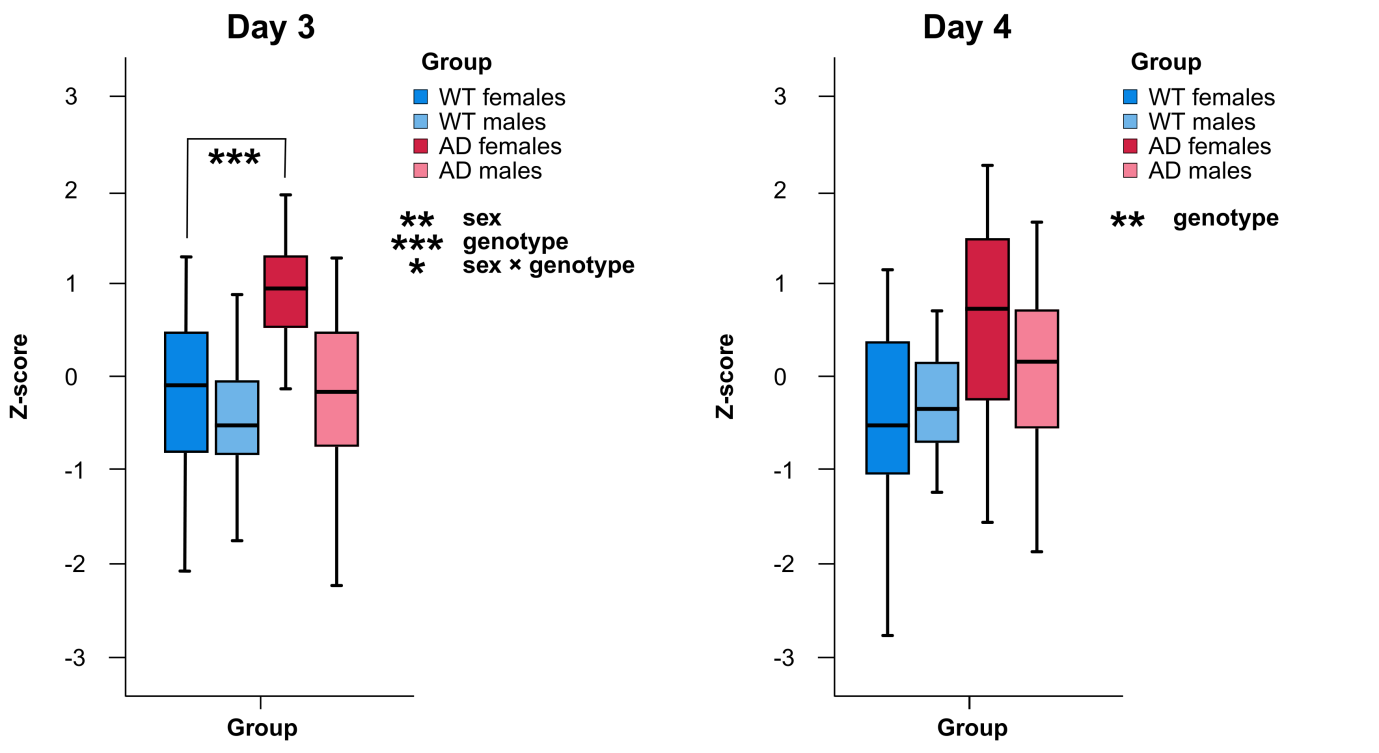
**

**Supplementary Table 1** Spatial navigation performance controlled for memory test scores

| **Controlled variable** | **Variable of interest** | **F** | ***P*** |
| --- | --- | --- | --- |
| AVLT 1-5 | Diagnosis (CN vs. AD aMCI) | 7.26 | 0.008 |
|  | SPS distance | 34.48 | <0.001 |
|  | Diagnosis * SPS distance | 0.38 | 0.540 |
| AVLT 30 | Diagnosis (CN vs. AD aMCI) | 7.59 | 0.006 |
|  | SPS distance | 34.44 | <0.001 |
|  | Diagnosis * SPS distance | 0.37 | 0.542 |
| Logical memory IR | Diagnosis (CN vs. AD aMCI) | 10.71 | 0.001 |
|  | SPS distance | 38.09 | <0.001 |
|  | Diagnosis * SPS distance | 0.39 | 0.535 |
| Logical memory DR | Diagnosis (CN vs. AD aMCI) | 10.43 | 0.001 |
|  | SPS distance | 38.16 | <0.001 |
|  | Diagnosis * SPS distance | 0.38 | 0.541 |
| ROCFT-R | Diagnosis (CN vs. AD aMCI) | 4.50 | 0.035 |
|  | SPS distance | 37.61 | <0.001 |
|  | Diagnosis * SPS distance | 0.33 | 0.566 |

*F* and *P* values refer to the main effect. CN, cognitively normal; AD aMCI, amnestic mild cognitive impairment with positive Alzheimer’s disease biomarkers; SPS distance, spatial pattern separation distance; AVLT 1-5, trials 1 to 5 total; AVLT 30, delayed word recall after 30 minutes; IR, Immediate Recall; DR, Delayed recall; ROCFT-R, Rey-Osterrieth Complex Figure Test – the Recall condition after 3 minutes.
